# Natal habitat imprinting counteracts the diversifying effects of phenotype-dependent dispersal in a spatially structured population

**DOI:** 10.1186/s12862-016-0724-y

**Published:** 2016-08-08

**Authors:** Carlos Camacho, David Canal, Jaime Potti

**Affiliations:** Department of Evolutionary Ecology, Estación Biológica de Doñana—CSIC, Av. Américo Vespucio s/n, 41092 Seville, Spain

**Keywords:** Cross-fostering, *Ficedula hypoleuca*, Local adaptation, Natal habitat preference induction, Matching habitat choice, Nonrandom dispersal, Pied flycatcher, Sympatric speciation

## Abstract

**Background:**

Habitat selection may have profound evolutionary consequences, but they strongly depend on the underlying preference mechanism, including genetically-determined, natal habitat and phenotype-dependent preferences. It is known that different mechanisms may operate at the same time, yet their relative contribution to population differentiation remains largely unexplored empirically mainly because of the difficulty of finding suitable study systems. Here, we investigate the role of early experience and genetic background in determining the outcome of settlement by pied flycatchers (*Ficedula hypoleuca*) breeding in two habitat patches between which dispersal and subsequent reproductive performance is influenced by phenotype (body size). For this, we conducted a cross-fostering experiment in a two-patch system: an oakwood and a conifer plantation separated by only 1 km.

**Results:**

Experimental birds mostly returned to breed in the forest patch where they were raised, whether it was that of their genetic or their foster parents, indicating that decisions on where to settle are determined by individuals’ experience in their natal site, rather than by their genetic background. Nevertheless, nearly a third (27.6 %) moved away from the rearing habitat and, as previously observed in unmanipulated individuals, dispersal between habitats was phenotype-dependent. Pied flycatchers breeding in the oak and the pine forests are differentiated by body size, and analyses of genetic variation at microsatellite loci now provide evidence of subtle genetic differentiation between the two populations. This suggests that phenotype-dependent dispersal may contribute to population structure despite the short distance and widespread exchange of birds between the study plots.

**Conclusions:**

Taken together, the current and previous findings that pied flycatchers do not always settle in the habitat to which they are best suited suggest that their strong tendency to return to the natal patch regardless of their body size might lead to maladaptive settlement decisions and thus constrain the potential of phenotype-dependent dispersal to promote microgeographic adaptation.

## Background

Selection of breeding environments is an important determinant of individual fitness and has therefore been a topic of considerable study in the realm of the ecology and evolution of dispersal [1–5]. Recent literature indicates that dispersal is typically nonrandom, and there is accumulating evidence that individuals often display a preference for a specific habitat type [6, 7]. Habitat preferences can have different underlying causes that include both genetic and environmental factors (e.g. [8–10]). First, experience in the natal patch can shape habitat preferences in adulthood, so that individuals tend to return to their birthplace or to other breeding habitats that resemble those they encountered at an early age, a phenomenon called natal habitat preference induction [11, 12]. Second, habitat preferences can be genetically determined [13, 14]. Finally, individuals may modify habitat selection according to their particular phenotype to settle in the habitats they are best suited to [15].

Numerous theoretical studies suggest that individual variation in habitat preferences can play an important role in population differentiation and ultimately also sympatric speciation (reviewed by [7]). Nevertheless, recent simulation-based studies of the evolutionary consequences of habitat preferences suggest that these may be largely contingent on the underlying preference mechanism [16, 17]. For example, natal habitat preference induction could promote population differentiation because the offspring of dispersers settling in a new, previously unused habitat may become reproductively isolated very quickly from the original source population. However, when the imprinting mechanism entails a substantial cost (e.g. energetic or nutritional costs of information processing and storing; [18]), population differentiation is more likely to occur through genetically-determined preferences [16]. It should be noted that different preference mechanisms may act redundantly in nature, producing similar dispersal patterns, and even operate synergistically to promote population differentiation [19]. For example, phenotype-dependent and natal habitat preferences may reinforce each other, since the former can facilitate local adaptation while the latter typically contributes to reproductive isolation [15, 19].

Different mechanisms generating habitat preference and nonrandom dispersal have been well characterized from a theoretical standpoint [6, 7, 11]. In addition, some efforts have been made to quantify the heritability of the propensity to disperse [14, 20] and of dispersal distance [14, 21] (but see [22]), and to assess the influence of early life experience [23–26] and phenotypic traits [27, 28] on dispersal and settlement decisions. However, almost no studies have been conducted to determine the relative contribution of different preference mechanisms to the expression of breeding site selection within a biologically realistic framework (but see [19]).

In this study, we tested the role of early life experience and genetic background in the settlement patterns of pied flycatchers (*Ficedula hypoleuca*) breeding in two contrasting habitats: a coniferous forest and a nearby deciduous forest separated by only 1 km. Males, and to a lesser degree, females breeding in the deciduous forest are morphologically different from those breeding in the nearby coniferous forest, the latter being smaller in size [28, 29]. Over 25 % of pied flycatchers returning to breed for the first time in the study area change habitats through dispersal and, from that moment, they rarely change the patch where they first bred [28]. Male pied flycatchers disperse between the two forests according to body size, so that individuals moving from the coniferous to the deciduous patch are larger than those moving the other way round, and also than those that remain in the coniferous patch. We have also shown that, contrary to the deciduous forest, where male size does not determine fitness, the latter increases non-linearly with body size in the coniferous forest. Nevertheless, the observed patterns of phenotype-dependent dispersal and settlement do not translate into fitness benefits [29]. Neither the proportion of individuals that return to their natal patch is what one would expect based on nest-site availability in each patch [28], suggesting that additional preference mechanisms (e.g. natal habitat preference induction or genetically-determined preference) must be operating.

Cross-fostering experiments between alternative habitats provide an essential tool to assess whether individuals settle to breed in one forest type or another owing to their genetic background or either mostly on the basis of the environment they experienced during sensitive periods along their ontogeny [30, 31]. Here, we capitalize on this approach to separate these two types of effects. Note that, since only one patch per habitat type is considered in this study, the terms “natal patch” and “natal habitat” are used interchangeably in the context of this work. No specific assessment is therefore made to separate the potential natal habitat effects on settlement decisions from those attributable to the specific features of each patch. As non-random, phenotype-dependent movements sustained across time may contribute to population structuring even at small spatial scales [27, 32, 33], we additionally explore whether there is detectable genetic divergence between pied flycatchers breeding in the two adjacent patches. Finally, we integrate our findings with earlier work on the same system concerning size-dependent dispersal and fitness differences among differently-sized individuals and discuss the relative importance of concurrent preference mechanisms in the spatial assortment of genotypes and phenotypes.

## Methods

### Study system

Fieldwork was conducted between 2006 and 2015 in a two-patch system: a mature oak (*Quercus pyrenaica*) forest of 9.3 ha, and a nearby (1.1 km) mixed pine (mostly *Pinus sylvestris*) plantation of 4.8 ha separated by unsuitable breeding habitat for pied flycatchers (i.e. a mixture of rock outcrops and riverside vegetation holding few suitable holes). Many aspects of the ecology of pied flycatchers have been studied in the deciduous and the coniferous forest since wooden nest-boxes (156 and 81) were provided in 1984 and 1988, respectively (e.g. [29]). Pied flycatchers are small (ca. 12 g) long-distance migrants overwintering south of the Sahara and breeding across Europe [34]. Males often arrive from spring migration before females, search for a suitable cavity for nesting and announce themselves to females by singing actively [34, 35]. Pied flycatchers exhibit strong natal and breeding site fidelity [36], and local recruitment rates are the highest reported in the literature on the species [37, 38]. Males, and to a lesser degree females, may postpone breeding until their second or, more rarely, third year of life [37].

### Field procedures

After arrival of the earliest males from spring migration, nest-boxes were checked at 1 − 3-day intervals to determine exact laying dates, clutch sizes, hatching dates and fledging success. Breeding birds were caught on day 8 (hatch day = 1) using a nest box trap, and measured for tarsus length (to the nearest 0.05 mm) and body mass (to the nearest 0.1 g). Fledgling mass and tarsus length were measured on day 13, when nestlings have already attained the definitive adult size [39, 40]. A small (<50 μL) blood sample was taken from the brachial vein of nestlings for molecular sexing and other genetic analyses (see e.g. [38]). Both adults and fledglings were individually marked with numbered metal rings. Nestling mortality was controlled from hatching to fledging by considering both the number of young surviving at day 13 and those found dead in the nest 18–20 days after hatching.

### Experimental procedures

Cross-fostering was conducted in 2006–2009 (complete clutches and half broods; 2–3 nestlings) and 2012–2013 (half broods; 2–3 nestlings). Note that recruits stemming from complete and half clutches or broods swapped between nests are equally suitable for the purpose of this study. We cross-fostered birds both within and between habitats in order to test, on one hand, how likely it is for a bird raised in its habitat of origin to return there to breed (i.e. control group) and, on the other hand, how likely it is for a bird experimentally raised in a habitat other than its habitat of origin to move back to its habitat of origin (i.e. experimental group). Cross-fostering was carried out 2–3 days after the onset of incubation (clutches) or on day 2–3 after hatching (broods). All transfers were performed between nests of similar phenology (i.e. matching hatching dates, and a difference of ± 1 day for eggs). Nestlings were individually marked for individual identification with a unique combination of colour markings on underparts (legs and wings) made with non-toxic felt pens and repainted every 2–3 days. To avoid skewing the masses of experimental nestlings in any direction during partial cross-fostering and thus minimize non-deliberate effects of competition between native and foster birds, all nestlings were weighed, ranked for weight, and then sequentially assigned to be either cross-fostered or remaining in the source nest.

Experimental manipulations had no apparent effect on recruitment rates (see ‘results’). For all recruits stemming from cross-fostering experiments that were subsequently captured as first-time breeders, we recorded whether they returned to breed in their rearing patch or, conversely, moved into the adjacent one. It should be noted that, because the two habitat patches sampled in this study are isolated from other patches by unsuitable habitat, pied flycatchers returning to breed in the study area must choose between settling in their natal or the adjacent plot, or risk not breeding at all [37].

### Genetic analyses

To examine genetic differences between pied flycatchers breeding in the two plots we followed the rationale described by [41]. Our dataset included 351 males and females breeding in 2005 and 2006 in the coniferous and the deciduous forest (108 and 243 birds, respectively), genotyped at 15 microsatellite loci (see [38]). Test for linkage disequilibrium were performed in Genepop 4.0 [42] and subsequently adjusted with Bonferroni corrections for multiples tests. We also used Genepop to test for deviation from Hardy-Weinberg equilibrium across loci and thus for the occurrence of inbreeding (Fis, estimated following [43]; Markov chain parameters were: 10,000 dememorisations, 1000 batches and 10,000 iterations per batch). Within each population, genetic diversity was calculated across loci using FSTAT [44] and compared using a Wilcoxon signed-rank test.

We used GENETIX to estimate the extent of genetic differentiation (*F*_ST_) between study plots (5000 permutations; [45]). Further, we tested for genotypic differentiation between populations in Genepop (“exact G test” option; Markov chain parameters were: 10,000 dememorisations, 1000 batches and 10,000 iterations per batch). Finally, we used a Bayesian approach as implemented in the program STRUCTURE [46] to infer the number of genetic clusters (K) in the population. Simulations were run assuming the admixture model with correlated allele frequencies. Four independent runs (*K* = 4), with twenty replicates for each K, were performed with a 10^5^ burning period followed by 10^6^ MCMC repeats after burning. To obtain the true value of K based on the method described by [47] we used the STRUCTURE HARVESTER web [48].

### Data analyses

To investigate the roles of early experience and genetic variation in determining selection of either habitat by adult pied flycatchers in the two-patch system, we fitted a generalized linear mixed model (GLMM; binomial error distribution and logit link function) including birds’ propensity to return to the rearing patch (0 = return to breed in the forest patch of their genetic parents; 1 = return to breed in the forest patch of their foster parents) as the response variable. As fixed effects, we included sex, the type of experimental treatment (cross-fostering within or between patches) as a control term, and the life stage of cross-fostering (egg or nestling) to account for possible ontogenetic effects on imprinting. Laying date at first reproduction, which is an accurate proxy for the arrival date in our study population [49], was also included as a covariate to account for the potential effect of arrival time (e.g. decreasing nest-site availability as the season progresses) on bird distribution across the two forests [50]. First-time breeders aged 2–3 years could have visited the study area in one or two seasons before being detected [51]. However, when restricting the analysis to birds breeding for the first time at age 1 (*n* = 43), results were exactly the same (details not shown), suggesting that the age at first breeding does not affect the settlement pattern of pied flycatchers.

It is possible that some fledglings had visited (and possibly imprinted on) the adjacent forest prior to departure to the wintering areas, since post-fledging explorations may exceed the minimum distance separating the two plots (see [52, 53]. As the direction of exploratory trips is assumed to be random [54], the likelihood of visiting the adjacent plot should increase with proximity of the latter to the rearing nest, thus obscuring the interpretation of our results. Accordingly, we used GPS coordinates to calculate the minimum linear distance between the rearing nest and the adjacent forest and included it in the model as a covariate.

Migratory birds may rely on a host of environmental cues and constraints to guide their settlement decisions after arrival, such as nest-site [55] and food availability [56], predation risk [57] or conspecific attraction [58]. Nest-site availability, measured as the annual number of nest-boxes not occupied by other hole-nesting species, differs between the two study patches and, furthermore, the magnitude of such differences may change over time [28]. Likewise, it is likely that other settlement cues, although not measured in this population, may vary over time and space, and this could severely bias the settlement decisions of pied flycatchers. To account for all these factors (i.e. between-year variability in habitat heterogeneity), we included in the model return year as a random effect. Nest identity was also fitted as a random effect to account for repeated measurements of experimental nests that produced ≥2 recruits (17 %, *n =* 99).

Finally, we tested the interaction between tarsus length and rearing patch according to the notion by [19] that, when phenotype-dependent dispersal occurs, an individual’s propensity to disperse into a non-natal habitat may depend on the interaction between its morphology and its rearing habitat.

GLMMs were fitted in R 2.14.0 (http://www.R-project.org) using the function *lmer* in the package ‘lme4’ [59]. Selection of the final model (containing only statistically significant terms) was carried out by dropping non-significant terms from a fully saturated model (containing all main effects and interactions) in a hierarchical way, starting with the least significant order terms.

## Results

Between 2006 and 2013, a total of 445 individuals from 116 nests (58 dyads) and 496 individuals from 120 nests (60 dyads) were cross-fostered between and within patches, respectively. Overall recruitment rates of cross-fostered young from experimental and control nests were, respectively, 14.7 % (57 recruits from 389 confirmed fledged offspring) and 11.8 % (48 recruits from 406 confirmed fledged offspring), the difference being not statistically significant (Pearson’s Chi-squared test with Yates’ continuity correction, χ^*2*^ = 1.152, *d.f*. = 1, *P* = 0.28). Recruitment rates did not differ between cross-fostered and non-manipulated fledglings either (respectively, 13.2 % (*n* = 795) and 11.7 % (*n =* 1658); χ^*2*^ = 1.003, *d.f*. = 1, *P* = 0.32), indicating that cross-fostering had no effect on the natural recruitment rates of pied flycatchers. Nests subjected to egg or nestling translocations showed similar recruitments rates (respectively, 13 % (*n =* 502 eggs) and 13.7 % (*n* = 293 nestlings); χ^*2*^ = 0.030, *d.f*. = 1, *P* = 0.83), indicating that the life stage at which the experiment was carried out did not bias our estimates. Overall recruitment rates did not differ either between cross-fostered males and females (respectively, 38 out of 335 vs. 44 out of 286 genetically-sexed nestlings; χ^*2*^ = 1.860, *d.f*. = 1, *P* = 0.17).

**Fig. 1:**
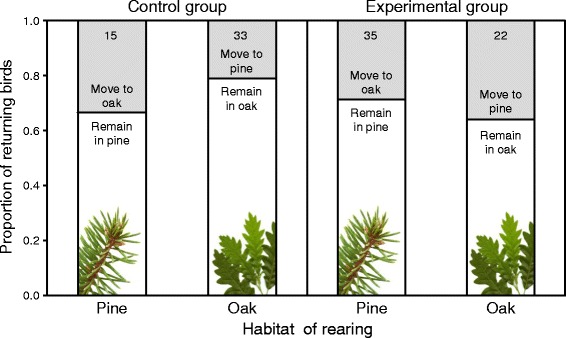
Proportions of pied flycatcher nestlings raised by foster parents that returned to breed in the same patch where they fledged (open area) or in the alternate habitat patch (*shaded area*) after being cross-fostered within (control group) or between (experimental group) patches. Figures inside bars are the total number of recruits (both sexes combined)

Most recruits from cross-fostered nests returned to breed in the forest patch where they had been transferred as nestlings or eggs, regardless of whether they had been cross-fostered within (75 %, *n* = 48 recruits) or between (70.2 %, *n* = 57) patches (Fig. 1; Table 1).

**Table 1:** Results of the GLMM (binomial error distribution and logit link function) analyzing the effects of the experimental treatment (cross-fostering within and between habitats), life stage of cross-fostering (egg and nestling), sex, proximity to the adjacent habitat patch, breeding date, rearing patch, body size (tarsus length) and the interaction between rearing patch and body size on birds’ propensity to return to the habitat where they had been raised (0 = return to breed in the non-rearing patch; 1 = return to breed in the rearing patch)

	Estimate	SE	*Z*	*P*
Intercept	0.963	0.218	4. 414	<0.001
Experimental treatment	0.243	0.442	0.550	0.582
Life stage of cross-fostering	0.214	0.456	0.470	0.638
Sex	−0.602	0.447	−1.345	0.179
Breeding date	0.012	0.055	0.228	0.819
Proximity to adjacent patch	0.000	0.001	1.083	0.279
Tarsus length	0.129	0.453	0.286	0.775
Rearing patch	−0.036	0.437	−0.083	0.934
Tarsus length x Rearing patch	−0.893	1.006	−0.888	0.375

Number of returning birds = 105; Number of years = 8; Number of nests = 83. Estimates and *P*-values of non-significant (removed) variables are from when they were added alone to a null model containing only the random effects

Results from the GLMM showed no effect of sex, breeding date, age at first reproduction, or proximity to the adjacent forest patch on birds’ propensity to return to the same patch where they were fostered (Table 1). The interaction between body size (tarsus length) and rearing patch had no statistically significant effect. Body size or patch effects alone were not influential either, indicating that there was no overall tendency for a particular phenotype to exchange between patches more than others, nor were individuals reared in one particular patch more likely to move into the adjacent patch (Table 1).

*F*_ST_ values revealed a low but statistically significant genetic differentiation between pied flycatchers breeding in the coniferous and the deciduous forest (*F*_*ST*_ = 0.002, *P* < 0.001), which was corroborated by the exact test of genotypic differentiation (χ^*2*^ = 53.99, *d.f*. = 30, *P* = 0.004). However, unlike in the coniferous habitat (χ^*2*^ = 22.82, *d.f*. = 30, *P* = 0.82), the deciduous forest population showed a deviation from H-W equilibrium (χ^*2*^ = 43.9, *d.f*. = 30, *P* = 0.05) due to an excess of homozygotes in three markers (*Fhy* 301, *Fhy* 339 and *Fhy* 401), which might affect the *F*_ST_ estimates. Further analyses showed that the excess of homozygotes in those loci was due to rare, private alleles appearing at low frequencies. After removing from the analyses the individuals carrying rare alleles in the deciduous patch, the two populations showed no evidence for H-W disequilibrium (deciduous patch: χ^*2*^ = 37.79, *d.f*. = 30, *P* = 0.15; coniferous patch: χ^*2*^ = 25.6, *d.f*. = 30, *P* = 0.69), had similar genetic diversity (*Z* = 0.78, *P* = 0.43) and, although marginally non-significant, *F*_*ST*_ values were of similar magnitude as that obtained from the entire data set (*F*_*ST*_ = 0.001, *P* = 0.06). These results suggest that rare alleles in some individuals from the deciduous forest likely contributed to, but were not fully responsible for, the genetic differentiation of pied flycatchers between the study plots. Bayesian clustering approach did not provide support for two genetic clusters. This is not surprising since, as shown by simulations [60], STRUCTURE may not appropriately summarize population structure in scenarios with a low number of genetic clusters (*n* = 2) and low genetic differentiation, as occurs in our system.

## Discussion

By following individuals that had been cross-fostered to the same or a different habitat patch type, we provided evidence that pied flycatchers returned mostly to breed as adults in the area where they had fledged, regardless of their sex and origin. This is consistent with the hypothesis that individuals’ decisions on where to settle are based on the environment they experienced at an early age, rather than on their genetic background. Birds breeding in the coniferous and the deciduous forest differ in body size [28, 29] and, as shown here, there is also evidence of genetic differentiation between the two populations. Combined with our previous work demonstrating that bird movement between the study plots is affected by body size [28], our results suggest that phenotype-dependent dispersal may act concurrently with natal site fidelity and thus contribute to the observed population differentiation of pied flycatchers over a very small geographic scale.

### Spatial and temporal scale of imprinting

Our study supports earlier evidence from experimental [61, 62] and observational studies [54] showing that pied flycatchers tend to return as adults to their natal patch, and that this is not genetically pre-programmed. However, it is not known whether the returning birds seek for a natal-like habitat or simply for their birthplace, as they only had one patch of each habitat type to choose between but, according to other studies on this species [61, 62], the former seems more likely. On the other hand, the observed propensity of pied flycatchers to settle in their natal patch could alternatively be interpreted in terms of spatial constraints − rather than choice − if, for example, nest-site availability away from the natal site is limited [50] or if it is easier to return to the same site after migration [63]. Nevertheless, it is unlikely that pied flycatchers are constrained to choose between the two study plots since, on one hand, the distance between them is extremely short, thus facilitating bird exchange between both sites and, on the other hand, nest-site availability does not yet seem to be a limiting factor for pied flycatchers in the study area [28, 29].

It is known that experience in the natal site can strongly influence later habitat choice in the pied flycatcher [54, 62], but evidence from translocation experiments suggests that imprinting may also occur well beyond fledging. [61] transferred caged 5–6 weeks old fledglings 250 km away from their birthplace in northern Germany and found that all recruits returned to the area where they had been released, not to their natal patch, indicating that imprinting may extend over the fledgling stage. However, such tendency to return to the site of release after translocation might alternatively result from the typically high costs of finding the way home [63].

Besides the duration of the imprinting process, the spatial scale of post-fledging exploration may affect settlement patterns in the subsequent years. Male pied flycatchers commonly settle closer to their natal sites than females, although both sexes seem to imprint on a similarly small area (i.e. several kilometres in diameter; [54]). Mean distance of post-fledging dispersal has been estimated between 0.6 − 1.4 km, depending on the study population [52, 53]. Exploratory trips by fledglings that extend beyond the distance separating the two forests (1.1 km) might facilitate imprinting on the patch adjacent to their natal patch, and thus determine future habitat shifts. However, even though detailed data on prospecting behaviour by fledglings are not available for the study area, this seems unlikely to bias our results, as the likelihood of changing habitats was apparently not determined by proximity of the rearing nest to the neighbour forest.

Some individuals could have visited the study area in one or more seasons before reproducing for the first time and explored future territories away from the area they explored as a fledgling [51]. But if this occurred, it certainly did not confound our estimates, as there were no differences between first-year and older first breeders in the propensity to change habitats. In addition, the timing of arrival from spring migration, which is known to have a profound effect on the breeding phenology and success of pied flycatchers [35], might influence territory acquisition. Late migrants could be time-constrained to choose their breeding site and thus occupy the remaining free territories, whatever the habitat type [50]. This may be particularly true for first-year breeders, which usually show delayed arrival dates [35, 37]. However, we found no phenological or age effects on birds’ propensity to return to their rearing patch. Finally, an additional potentially confounding factor is that some individuals could perceive certain forest patches as offering better foraging and nest-site opportunities than others based on e.g. asymmetry in breeding density and presence of competitors, and settle accordingly [27]. However, we think this is unlikely to bias our estimates for two main reasons. First, as shown here, males and females are equally likely to return to breed in the area where they were raised, despite the fact that only the former compete for nest sites [34]. Second, earlier results on the same system indicate that nest-site availability and population density do not play an important role in the local distribution of pied flycatchers [28, 29].

### Natal habitat imprinting vs. phenotype-dependent dispersal

So far, few studies have attempted to tease apart the relative effects of natal patch preferences and phenotype-dependent dispersal on population differentiation. [19] also reported a strong tendency in lake and stream sticklebacks to return to their natal area and, as in pied flycatchers, their movement between habitats was phenotype-dependent. In our study population, around a quarter of the (unmanipulated) natal dispersers do not return to the forest patch they presumably imprinted on. Conversely, they move naturally into the adjacent habitat patch according to their body size, so that individuals dispersing from the coniferous to the deciduous forest are larger than those moving the other way round (respectively, 19.47 ± 0.07 mm and 19.39 ± 0.08 mm; mean ± SE; [28]). Likewise, we observed qualitatively similar differences between cross-fostered individuals moving from the coniferous to the deciduous forest and those moving the other way round (respectively, 19.52 ± 0.08 mm and 19.28 ± 0.15 mm; mean ± SE). Even though this difference (1.1 %) might seem modest, it is well within the range reported in other studies made at small spatial scales (5–10 km; 0.9–1.2 % difference; [33, 64, 65]. Nevertheless, in contrast to the large sample of unmanipulated individuals, the effect of the interaction between body size and rearing patch on the propensity to change habitats did not reach significance in our experimental birds (see ‘results’), possibly because the sample size of ‘non-philopatric’ recruits stemming from the experiment is very limited compared with the observational study (29 vs. 115). Indeed, a power analysis based on the observed differences between the two groups of dispersers (effect size *d* = 0.52) showed that, for α = 0.05, an overall sample size of *n* = 120 would be required to have 80 % power, which means that, according to the actual recruitment rate (13.2 %) and proportion of recruits moving between habitats (27.6 %), over 3200 individuals would need to be cross-fostered. Still, taken together, the results of this experiment and our previous studies [28, 29] give support to the notion by [19] that phenotype-dependent dispersal and natal site fidelity may act concurrently.

More pied flycatchers disperse between the two habitats compared with the sticklebacks study (30 % vs. 10 %) but, unlike in the stickleback populations, phenotype-dependent dispersal and natal habitat preferences of pied flycatchers do not appear to act synergistically but partially cancel out each other. [19] showed that morphologically different sticklebacks preferentially settle in the habitat conferring a fitness advantage (ultimately promoting microgeographic adaptation). By contrast, decisions on where to settle and fitness appear to be decoupled in our study population [29], possibly because of the strong tendency to return to the natal habitat patch, not to that in which their particular phenotype performs best (see [26, 66]; but see [12]. Thus, the strong (maladaptive) reluctance of the majority of birds to move away from either of the two habitat patches might constrain, rather than promote, microgeographic adaptation and population divergence. Even with this constraint, the level of genetic differentiation between both pied flycatcher populations is of the same order of magnitude as that reported between the less mobile lake and stream sticklebacks (*F*_ST_ = 0.008; [19]), and also similar to those found over much more extensive regions in other highly mobile organisms, such as the house sparrow (*Passer domesticus*) (*F*_ST_ = 0.004 across Finland; [67]) or the Eurasian reed warblers (*Acrocephalus scirpaceus*) (*F*_ST_ = 0.013 across Europe; [68]). Accordingly, it seems that the diversifying effects of phenotype-dependent dispersal alone might be strong enough to generate a detectable signal of population differentiation at exceedingly small geographic scales [27, 33].

## Conclusions

Early experience in the natal patch may play a crucial role in determining subsequent dispersal and settlement in the pied flycatcher ([54, 61, 69], this study) and possibly also in a broad range of animals [11]. Taken together, the results presented herein support previous evidence indicating that phenotype-dependent dispersal between the two plots might contribute to the observed morphological [28] and genetic (this study) differentiation of pied flycatchers. However, the potential of phenotype-dependent dispersal to increase the magnitude of divergence between these two populations might be constrained by the strong natal site fidelity, as individual performance in each forest patch is strongly determined by morphology [29]. We suggest that the heretofore largely neglected − but likely widespread − interplay between early experience in the natal site and individual phenotype should be fully taken into account in future studies investigating the mechanisms underlying non-random dispersal and habitat selection.
